# Freestanding Nitrogen‐Doped Carbons with Hierarchical Porosity for Environmental Applications: A Green Templating Route with Bio‐Based Precursors

**DOI:** 10.1002/gch2.202100062

**Published:** 2021-08-19

**Authors:** Mojtaba Mohseni, Nikolai Utsch, Christian Marcks, Kristof Demeestere, Gijs Du Laing, Süleyman Yüce, Robert G. Keller, Matthias Wessling

**Affiliations:** ^1^ Aachener Verfahrenstechnik ‐ Chemical Process Engineering RWTH Aachen University Forckenbeckstr. 51 52074 Aachen Germany; ^2^ Department of Green Chemistry and Technology Ghent University Coupure Links 653 Ghent 9000 Belgium; ^3^ DWI ‐ Leibniz Institute for Interactive Materials Forckenbeckstr. 50 52074 Aachen Germany

**Keywords:** adsorption, chitosan‐based carbons, hard‐templating, mesoporous materials, microporous materials, oxygen reduction reaction

## Abstract

Powdery hierarchical porous carbons serve as cost‐effective, functional materials in various fields, namely energy storage, heterogeneous catalysis, electrochemistry, and water/wastewater treatment. Such powdered activated carbons (PAC) limit new module designs and require further preparation steps, for example, adding polymeric binders, to be shaped into a standalone geometry. Polymeric binders, however, can block PACs’ catalytic and active sites and, more importantly, pose the risk of secondary pollution for environmental purposes, especially in the context of clean water supply. This study introduces a novel synthesis method for fabricating freestanding nitrogen‐doped carbons with hierarchical porosity using chitosan and sucrose as green precursors. Chitosan supplies nitrogen and acts as a backbone, giving a freestanding geometry to the final product, and sucrose is a carbon‐rich precursor. The proposed method employs ice‐ and hard‐templating for macropores and mesopores and combines carbonization and activation steps with no required activating agent. Final freestanding carbons function as adsorbents for removing persistent pollutants, as binder‐free electrodes with high specific surface area and capacitive current, and as tubular gas diffusion electrodes for oxygen reduction reactions. These freestanding carbons enable new module designs and can be scaled‐up by numbering‐up, serving as bio‐based functional materials for a wide range of applications involving porous heteroatom‐doped carbons.

## Graphical abstract

A green templating route to produce tailor‐made monolithic carbons using chitosan and sucrose is herein reported. The synthesis method offers high flexibility to tune inner porosity and surface area, heteroatom content, and the final carbon monolith's geometry. Additionally, the monolithic form with adequate separation properties paves the way towards more sustainable concepts and novel module designs.

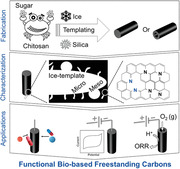

## 1 Introduction

Porous carbons are characterized by a large specific surface area, high porosity, and corrosion resistance as well as thermal and electrical conductivity. They serve as functional materials in a wide range of applications, namely energy storage and batteries,^[^ ^1^ ^]^ electro‐catalysis,^[^ ^2^ ^]^ hydrogen storage systems,^[^ ^3^ ^]^ and water/wastewater treatment.^[^ ^4^, ^5^, ^6^ ^]^ Although most of the reported hierarchically porous carbons are powdery or were applied as powdered materials for the final use, monolithic carbons with tunable porosity and heteroatom content are of particular interest from an engineering point of view. A carbon monolith can directly be exploited as freestanding material, e.g., an electrode,^[^ ^7^ ^]^ an adsorbent,^[^ ^8^ ^]^ and both^[^ ^9^ ^]^ in a module.

In contrast, powdered carbons need to i) be mixed with a polymeric binder and ii) pressed on a rigid support for physical stability to make standalone geometries. The former could block the active sites and porosity of carbons by coverage, and the latter may destroy carbon's hierarchical porosity by pressure, leading to a deficient use of carbon materials. Additionally, using polymeric binders for environmental applications like capacitive deionization, electrochemical advanced oxidation processes, and adsorption to supply clean water should be avoided due to secondary pollution concerns^[^ ^10^ ^]^ by active layer fall‐off because of mechanical wearing.^[^ ^11^ ^]^ Besides, to the best of our knowledge, all the hierarchically porous carbons reported in the literature were applied in a planar geometry for their final application, whereas (micro)tubular geometries offer higher surface to volume ratios,^[^ ^12^, ^13^ ^]^ larger packing densities,^[^ ^12^, ^13^ ^]^ and new module designs for different purposes.^[^ ^8^, ^9^, ^13^, ^14^, ^15^ ^]^

Acid‐catalyzed sol‐gel polymerization of resorcinol and formaldehyde has been extensively studied and reported for the synthesis of hierarchically porous carbon monoliths, offering extremely high surface areas up to 3000 m^2^ g^−1^.^[^ ^16^, ^17^ ^]^ Researchers have also reported synthesizing monolithic carbons using monolithic silica as a porous and interconnected mold for carbon precursors, e.g., sucrose, producing a negative replica of the porous silica monolith,^[^ ^18^ ^]^ which is chemical‐ and time‐consuming. Alternatively, Antonietti's group introduced hydrothermal carbonization (HTC) as a greener and more environmentally friendly method than the conventional sol‐gel process for producing monolithic carbons, where inexpensive biomass‐derived compounds, e.g., carbohydrates, are utilized.^[^ ^19^, ^20^, ^21^, ^22^ ^]^

Nonetheless, carbons after HTC represent low porosity and inner surface area and must be further treated at high temperatures (500–900 °C) for activation.^[^ ^17^, ^18^, ^23^, ^24^, ^25^ ^]^ Besides, the HTC‐derived monolithic carbons primarily lack macroporosity, which is imperative for enhanced mass‐transfer towards inner parts of carbon monoliths. One green technique for introducing such macropores is ice templating or freeze‐casting, where water serves as the porogen.^[^ ^16^, ^26^, ^27^ ^]^ The macropore's direction and size can be controlled by the freezing rate.^[^ ^16^ ^]^ Freeze‐casting has also been widely reported for fabrication of 3D monolithic carbons for different applications, e.g., adsorption,^[^ ^28^, ^29^ ^]^ oil/water separation,^[^ ^29^, ^30^ ^]^ and tissue engineering as well as nanocarries.^[^ ^31^, ^32^ ^]^ However, these studies have used commercial carbon nanotubes or graphene oxide as carbon materials instead of exploiting bio‐based and renewable resources. Estevez and co‐workers^[^ ^33^ ^]^ have introduced a monolithic porous carbon using glucose as the precursor and applying ice‐ and silica‐templating. Yet, the monolithic carbon was ground and mixed with a binder for electrochemical applications.^[^ ^33^ ^]^ Therefore, novel methods using green templating routes and bio‐based precursors are demanded to synthesize hierarchically porous carbon monoliths with direct implementation as electrodes and adsorbents. Such a carbon monolith could shorten the incubation time between newly developed materials and final module design and construction. More importantly, it would preclude secondary pollution caused by polymeric binders for environmental applications, especially water/wastewater treatment.

**Figure 1:**
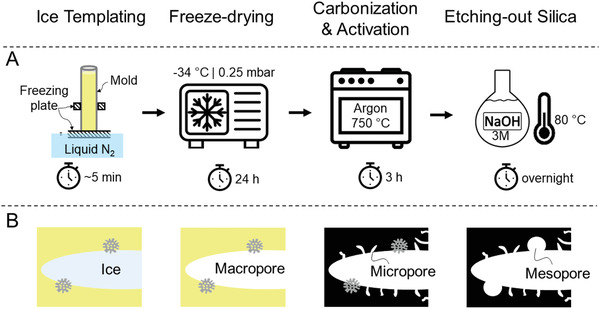
Schematic of all fabrication steps used for making freestanding carbons in this study (A), and the formation of hierarchical porosity during these steps (B).

**Figure 2:**
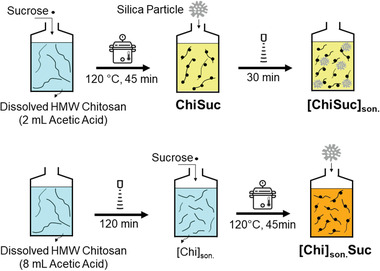
Schematic of the preparation methods for three different precursors with the same amount of high molecular weight (HMW) chitosan (in short: Chi), sucrose (in short: Suc), and silica. []_son._ indicates the sonicated solution, if applicable.

This study introduces a novel approach to produce monolithic carbons with a hierarchical porosity and a freestanding geometry using two bio‐based precursors: chitosan and sucrose. Chitosan acts as a nitrogen source and backbone, giving the final product a freestanding shape, while sucrose is a carbon‐rich precursor for higher carbonization yield. Hydrolyzed sucrose at 120 °C reacts with free amino groups of chitosan through the so‐called “Maillard” chemistry during 15 min reaction time, being much shorter than the HTC approach (t ≥ 5 h).^[^ ^19^, ^20^, ^21^, ^22^ ^]^ The resulting yellowish solution goes through ice‐ (macroporosity) and silica‐templating (mesoporosity) and becomes carbonized and activated (microporosity) in one step. **Figure** **1** displays a scheme of fabrication steps. Also, the effect of the precursors’ sonication (**Figure** **2**) on the ice‐templated macroporous network and textural properties of final products was investigated. As a first environmental application, monolithic carbons were used as freestanding adsorbents for removing the antibiotic sulfamethoxazole (SMX), a frequently detected micropollutant in water. Furthermore, we demonstrate the monolithic carbons’ functionality as cylindrical electrodes and tubular gas diffusion electrodes (GDEs) for electrochemical applications without adding any polymeric binders, illustrating the method's flexibility in tailoring pore size, pore direction, and geometry of final products.

## 2 Results and Discussion

### 2.1 Characterization of Carbons: Effect of Sonication

**Figure 3:**
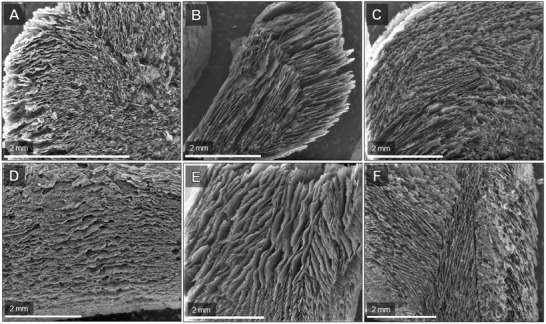
SEM images of ChiSuc (A,D), [ChiSuc]_son._ (B,E) with 30 min sonication shortly before freezing, and [Chi]_son._Suc (C & F) with 120‐min‐sonicated chitosan before autoclaving. (A), (B), and (C) represent the cross‐section of monolithic carbons, which is perpendicular to the freezing direction; while (D), (E), and (F) represent the peripheral surface of monolithic carbons, which is parallel to the freezing direction (Figure 1A).

The effect of sonication was assessed using three different recipes (ChiSuc, [ChiSuc]_son._, and [Chi]_son._Suc) with the same amount of chitosan and sucrose as precursors (Figure 2). **Figure** **3** shows the scanning electron microscopy (SEM) images of as‐synthesized carbons in polypropylene (PP) molds for both cross‐section and peripheral surfaces. Generally, a longitudinal freezing direction with no noticeable radial pattern can be observed for all three recipes as expected because of thermally non‐conductive PP molds. The cross‐section of ChiSuc predominantly consists of small pores, which are isolated from the adjacent ones (Figure 3A). In contrast, [ChiSuc]_son._ represents a lamellar structure of macropores, which are much bigger and stretched all over the cross‐section (Figure 3B). In comparison, [Chi]_son._Suc shows a lamellar structure like [ChiSuc]_son._ but with smaller widths and shorter lengths (Figure 3C). The difference in macropores observed between the carbons can be attributed to the precursor's molecular size, as freezing conditions remained similar for all. Gutiérrez and co‐workers have illustrated the impact of the precursor's molecular weight (MW) on the final ice‐templated pore sizes.^[^ ^34^ ^]^ It has been illustrated that the increase of MW (13 000 to 130 000 g mol^−1^) changes the shape of ice‐templated macropores and their size from wide and long pores to narrow and short ones at the same freezing rate.^[^ ^34^ ^]^ This relates to the extent of ice crystals’ growth in solutions, which, in turn, depends on the adsorption and desorption of solute molecules on the ice crystals.^[^ ^34^ ^]^ When a solute molecule irreversibly adsorbs on an ice crystal's surface and is sufficiently big to thoroughly cover it, ice crystal growth becomes limited and stops, resulting in narrow and isolated pores after freeze‐casting. Undoubtedly, sonication of the chitosan stock solution ([Chi]_son._Suc) or the precursor ([ChiSuc]_son._) decreases the MW of solutes before freezing.^[^ ^35^ ^]^ This impact has been more substantial for [ChiSuc]_son._ since sonication occurred shortly before the freezing step, resulting in the largest macropores because of sufficient ice crystals’ growth. A similar trend is also observable on the peripheral images (Figure 3D–F): longitudinal pores along the carbon monolith with different pore sizes, as discussed above. It is worth mentioning that open and interconnected macropores on the peripheral surface accelerate the liquid ingress towards monoliths’ inner parts, which ChiSuc severely lacks based on Figure 3D.

**Figure 4:**
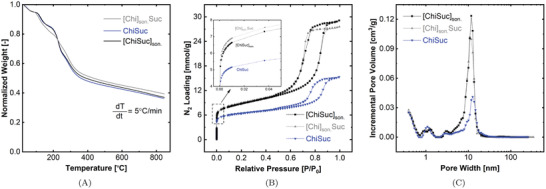
Thermogravimetric analysis (TGA) (A) and N_2_‐physisorption analysis (B & C) of ChiSuc, [ChiSuc]_son._, and [Chi]_son._Suc. A) carbonization of green composites under N_2_ gas from 30 °C to 850 °C; B) N_2_‐adsorption/desorption isotherms of final carbons at 77 K; C) corresponding pore size distribution (PSD) based on the DFT model for slit‐shaped pores.

The carbonization of ChiSuc, [ChiSuc]_son._, and [Chi]_son._Suc composites was monitored using thermogravimetric analysis (TGA) under a dynamic N_2_ atmosphere, represented in **Figure** **4**A. Carbonization yield is defined as the remaining mass of final carbons to the initial mass of precursors achieved at the end of the TGA analysis. Generally, the course of mass loss for ChiSuc and [ChiSuc]_son._ is remarkably similar while they slightly differ from [Chi]_son._Suc. Observing no difference between ChiSuc and [ChiSuc]_son._ implies that the sonication after autoclaving has not caused any further reaction between chitosan and sucrose as aimed. The first mass drop observed for all samples occurred between 90 and 140 °C resulting from the evaporation of physically adsorbed water followed by the release of hydrogen‐bonded water.^[^ ^36^ ^]^ The second drop lies between 185 and 240 °C, which is much steeper for ChiSuc and [ChiSuc]_son._ compared to [Chi]_son._Suc. Rybarczyk et al.^[^ ^36^ ^]^ have reported that the pure chitosan has a mass loss during carbonization at 195 °C because of further dehydration or preliminary deacetylation of chitosan without degrading the main chitosan chain. As described in the experimental section and depicted in Figure 2, the chitosan stock solution for [Chi]_son._Suc contained four times more acetic acid and was sonicated for 120 min before adding sucrose and being placed in an autoclave. Both sonication and increased acetic acid content promote the degree of deacetylation in chitosan.^[^ ^35^ ^]^ The higher the degree of deacetylation, the higher the number of free amino groups, and the faster the reaction between amino groups and sugar (Maillard chemistry), as can be noticed based on the evolved color after autoclaving (Figure S1, Supporting Information).^[^ ^37^, ^38^ ^]^ Thus, [Chi]_son._Suc contained fewer acetyl groups compared to ChiSuc and [ChiSuc]_son._ to be deacetylated during the carbonization at 195 °C. This lower mass loss of [Chi]_son._Suc between 185 and 240 °C could explain the slightly higher carbonization yield (γ = 0.39 ± 0.01 ) compared to ChiSuc (γ = 0.36 ± 0.01) and [ChiSuc]_son._ (γ = 0.37 ± 0.02). However, the dissimilarity between carbonization yields partly lies within the experimental error.

Figure 4B shows N_2_‐physisorption isotherms for final carbon products. Based on IUPAC standards, the depicted graphs all show a combination of type one (I) and four (IV) isotherms.^[^ ^39^ ^]^

Type one represents the characteristics of microporous materials in which a steep uptake is observable at very low N_2_ relative pressures (P/P_0_) because of the strong interactions between adsorbate and adsorbent. This finite uptake value is controlled by the accessible volume of micropores,^[^ ^39^ ^]^ which are forming during the carbonization while inherently less stable functional groups leave the carbon surface.^[^ ^40^, ^41^ ^]^ In this work, the carbon surface has increased due to the ice‐templating, and micropores form on the inner surface of ice‐templated macropores (Figure 1B). Hence, the amount of micropores detected depends on two factors: the size of ice‐templated macropores and the accessibility of the 3D pore network. The smaller the ice‐templated pores, the higher the external surface on which micropores may form; and, the more interconnected the 3D pore network, the less restricted the accessibility to the formed micropores. On the one hand, the latter factor prevails for [ChiSuc]_son._ compared to ChiSuc, leading to a higher nitrogen loading (Figure 4B) at low relative pressures despite having much wider pore sizes (Figure 3A,B). In other words, the ice‐templated pores in ChiSuc are isolated, and micropores formed on them are not accessible during the N_2_‐physisorption. On the other hand, [Chi]_son._Suc shows even a slightly higher nitrogen loading than [ChiSuc]_son._ (inset graph of Figure 4B) while losing less mass during carbonization (Figure 4A). This can be attributed to the smaller size of macropores in [Chi]_son._Suc than [ChiSuc]_son._ with good accessibility (finer lamellar structure than [ChiSuc]_son._ (Figure 3B,C). As shown in **Table** **1**, the average of micropore volume is 0.15, 0.17, and 0.18 cm^3^ g^−1^ for ChiSuc, [ChiSuc]_son._,and [Chi]_son._Suc, respectively. The raw data for the cumulative pore volume of fabricated carbons at different pore sizes, as calculated based on the DFT model, are presented in Table S1, Supporting Information.

**Table 1:** Structural properties and elemental composition of ChiSuc, [ChiSuc]_son._, and [Chi]_son._Suc through N_2_‐physisorption (*n* = 2) and XPS (*n* = 3) analyses, respectively

Carbon	N_2_‐physisorption			XPS [wt%]		
	S_BET_ [m^2^ g^−1^]	V_total_ [cm^3^ g^−1^]	V_micro_ [cm^3^ g^−1^]	C	O	N
ChiSuc	524 ± 1 2	0.48 ± 0.01	0.15	88.1 ± 1.0	8.6 ± 0.4	2.5 ± 0.9
[ChiSuc]_son._	684 ± 5	0.93	0.17	89.4 ± 1.2	8.8 ± 2.9	2.7 ± 0.6
[Chi]_son._Suc	703 ± 6	0.88 ± 0.01	0.18	88.1 ± 1.5	8.0 ± 1.0	3.4 ± 0.2

Type four of isotherms characterizes mesoporous materials with pores wider than 4 nm given the accompanied hysteresis.^[^ ^39^ ^]^ Thus, all three carbons are mesoporous, resulting from the silica‐templating with a peak centered at 11.7 ± 0.9 nm (Figure 4C). Interestingly, despite adding the same silica amount in all recipes, the peak's height significantly differs between sonicated ones ([ChiSuc]_son._ and [Chi]_son._Suc) and ChiSuc. The minimum value was achieved for ChiSuc, followed by [Chi]_son._Suc and [ChiSuc]_son._. This means that less silica was removed during the etching‐out step for ChiSuc compared to [Chi]_son._Suc and [ChiSuc]_son._. TGA results under a dynamic O_2_ atmosphere support this explanation (Figure S3, Supporting Information). The remaining relative mass after burning‐out of carbon is 0.09 for ChiSuc while being negligible for both [ChiSuc]_son._ and [Chi]_son._Suc, which indicates the presence of thermally stable silica particles in ChiSuc after the etching‐out process. As mentioned above, ChiSuc severely lacks open macropores and an interconnected 3D pore network, clearly visible in Figure 3D, impairing deep penetration of NaOH into the monolith during the etching‐out step. The average of total pore volume is 0.48 cm^3^ g^−1^ for ChiSuc and increases to 0.88 and 0.93 cm^3^ g^−1^ for [Chi]_son._Suc and [ChiSuc]_son._, respectively, because of higher mesoporosity.

The corresponding specific surface area for ChiSuc reaches 524 ± 12  m^2^ g^−1^ and increases to 684 ± 5 m^2^ g^−1^ for [ChiSuc]_son._. As expected, the highest surface area (703 ± 6 m^2^ g^−1^) has been achieved for [Chi]_son._Suc as it shows the highest micropore volume among others. These BET values stand out for monolithic carbons provided that no additional activation step was employed during the synthesis. For instance, Guo and co‐workers^[^ ^42^ ^]^ used chitosan as a precursor and Urchin‐like silica as a template to make 3D hierarchically porous carbon powders through HTC with an additional thermal activation. The BET surface area reported varies from 482 m^2^ g^−1^ to 873 m^2^ g^−1^ depending on the recipe.^[^ ^42^ ^]^ Estevez et al.^[^ ^33^ ^]^ selected glucose as a precursor to synthesize monolithic carbons and tuned the porosity using the silica content. The final product was mesoporous with a BET of 841 m^2^ g^−1^. However, the carbon lacked microporosity and needed an activation step. Also, a higher silica content (precursor: silica = 1:1) than in this study (= 2:1) was used.^[^ ^33^ ^]^

XPS results revealed that all three carbons contain nitrogen as the heteroatom, represented in Table 1. Accordingly, the detected surface N‐content is 2.5 ± 0.9 wt% , 2.7 ± 0.6 wt%, and 3.4 ± 0.2 wt% for ChiSuc, [ChiSuc]_son._, and [Chi]_son._Suc, respectively. Although the difference between N‐content values mainly lies within the experimental error, the highest average value of [Chi]_son._Suc with the lowest standard deviation is noticeable among others. This could be associated with the promoted Maillard reaction during autoclaving based on the solution color (Figure S1, Supporting Information).^[^ ^37^ ^]^. High resolution N 1(s) analysis shows two distinct binding energies for all recipes (Table S2, Supporting Information): one at 398.5 ± 0.1 eV with 36.4 ± 4.7% area, which is assigned to pyridinic groups;^[^ ^40^, ^43^, ^44^ ^]^ another at 401 ± 0.1 eV with 63.6 ± 4.7% area, which is assigned to quaternary or graphitic‐N.^[^ ^40^, ^43^, ^44^ ^]^ A high share of graphitic‐N in the monolithic carbons results from the high carbonization temperature (750 °C) and favors micropollutants adsorption^[^ ^45^, ^46^ ^]^ as well as electrocatalytic applications.^[^ ^42^, ^44^ ^]^

### 2.2 Monolithic Porous Carbons as Adsorbents

**Figure 5:**
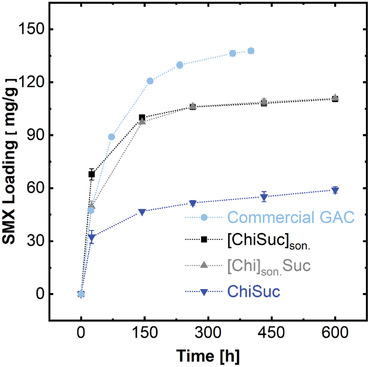
Monolithic porous carbons and commercial GAC as adsorbents: sulfamethoxazole (SMX) adsorption on ChiSuc, [ChiSuc]_son._, [Chi]_son._Suc, and commercial GAC at room temperature, [SMX]_0_ = 25 ± 1 mg L^−1^, [pH]_0_ = 5.1. Each point is the average of triplicate with standard deviation as error bars.

**Figure** **5** depicts the SMX adsorption capacity of three carbons over time. A set of adsorption experiments under similar conditions was also conducted using a granular activated carbon (Charcoal GAC, 2.5 mm) for comparability to commercial products. Based on Figure 5, one could assume that the adsorption equilibrium has been nearly reached after 250 h as the adsorption rate significantly decreases afterwards. The experimental data were well fitted (Figure S4, Supporting Information, R^2^ ≥ 0.97) to the pseudo‐first‐order kinetic model,^[^ ^48^, ^49^ ^]^ and fitting parameters are listed in Table S3, Supporting Information. Accordingly, the equilibrium capacity for ChiSuc, [ChiSuc]_son._, and [Chi]_son._Suc is 53 ± 2, 106 ± 2, and 107 ± 2 mg g^−1^, respectively. Since as‐synthesized adsorbents represent nearly similar surface chemistry (Table 1), one could associate the difference in the equilibrium capacity to the BET surface area of the adsorbents. However, BET solely cannot explain the double amount of equilibrium capacity achieved for sonicated samples because [ChiSuc]_son._ and [Chi]_son._Suc only hold around a 1.3 times higher BET surface area. Hence, it can be deduced that SMX has not accessed the whole BET surface area of ChiSuc during the adsorption experiment because of isolated macropores. However, the rate constant of the pseudo‐first‐order adsorption is almost similar for both ChiSuc (0.036 ± 0.009  h^−1^) and [ChiSuc]_son._ (0.040 ± 0.004 h^−1^), being higher than the one for [Chi]_son._Suc (0.023 ± 0.003  h^−1^). The lower rate constant of [Chi]_son._Suc compared with [ChiSuc]_son._ having similar equilibrium capacities (Table S3, Supporting Information) can be attributed to its finer ice‐templated macropores (compare Figure 3B,C).

In comparison to monolithic carbons, commercial GAC represents the highest equilibrium capacity of 135 ± 2 mg g^−1^, which is associated with its significantly larger BET of 1355 m^2^ g^−1^ (Table S3, Supporting Information). Nevertheless, an increase of approximately 94% BET for GAC only led to around 27% higher equilibrium capacity, implying that most parts of GAC specific surface area have remained unused after the adsorption experiment. Additionally, the commercial GAC, despite having smaller dimensions than as‐synthesized monolithic carbons, represents the lowest adsorption rate constant of 0.016 ± 0.001  h^−1^ because of its microporous characterization (Table S3, Supporting Information) and lack of macropores for better solution ingress. Thus, as‐synthesized monolithic carbons, besides suggesting comparable SMX adsorption capacities (ChiSuc]_son._ and [Chi]_son._Suc) to commercial GAC, offer faster kinetics, which is crucial from an engineering point of view for module design.

### 2.3 Monolithic Porous Carbons as Electrodes

**Figure 6:**
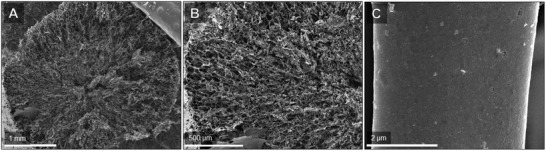
FESEM images for ChiSuc recipe frozen in a brass mold and carbonized at 1000 °C. A and B: cross‐section of monolith with apparent radial freezing; C: magnified image of the inner carbon structure on which mesopores are templated.

The tubular housing for the ice‐templating can be used to engineer the macropores’ direction. Changing the mold's material from thermally non‐conductive PP (Figure S2, Supporting Information) to conductive brass one (Figure S5A, Supporting Information), a radial macroporous pattern is templated. **Figure** **6** represents field emission electron microscopy (FESEM) images of ChiSuc frozen in a brass mold and carbonized at 1000 °C (ChiSuc_Br_1000). It has to be mentioned that fabrication of sonicated precursors ([ChiSuc]_son._ and [Chi]_son._Suc) in brass molds was not pursued because, after lyophilization, the composites could not detach from the molds. Compared to PP mold (Figure 3A), a radial freezing toward the center of carbon can be observed (Figure 6A,B). The ice‐templated pores are smaller because of the faster freezing rate along the mold's length, which is induced by the conductive mold and its smaller inner diameter (ID_Brass_ = 7 mm vs ID_*PP*_ = 10 mm). Pores in the center are smaller, and material density increases as the solution is pushed away by symmetrical radial freezing, being accumulated in the center. Figure 6C represents the mesopores formed on the surface of the ice‐templated macropores’ walls, showing the pore structure's hierarchy.

N_2_‐physisorption analysis revealed that ChiSuc_Br_1000 has a BET specific surface area, micropore volume, and total pore volume of 629 m^2^ g^−1^, 0.2 cm^3^ g^−1^, and 0.3 cm^3^ g^−1^, respectively (Table S4, Supporting Information). The higher micropore volume relates to smaller ice‐templated macropores, as discussed earlier, and higher carbonization temperature.^[^ ^40^ ^]^ It has to be noted that the faster freezing rate induced by the brass mold also builds up a dense 3D pore network, given the fast ice nuclei formation and their limited growth. Consequently, ChiSuc_Br_1000 possesses much smaller pore volume compared to ChiSuc (V_total_ = 0.48 cm^3^ g^−1^), resulting from the impaired silica removal during the etching‐out step as evidenced by the low peak at 11.7 ± 0.9 nm (Figure S6, Supporting Information). Thus, ChiSuc_Br_1000 is more microporous (67% of V_total_) than ChiSuc (30% of V_total_) despite having the same precursors (ChiSuc) and silica content. This clearly shows the versatility of the presented synthesis method in tailoring textural properties of final products.

**Figure 7:**
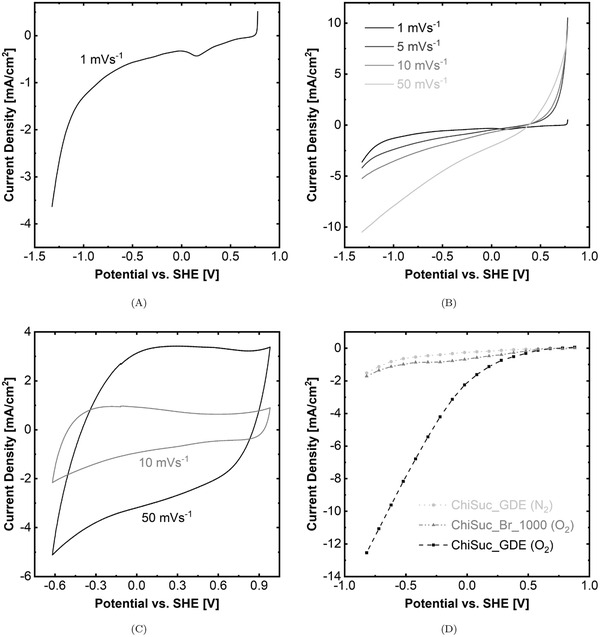
Functional monolithic and tubular carbons as electrodes. A,B) Linear sweep voltammograms (LSV) and C: Cyclic Voltammograms (CV) of ChiSuc, frozen in a brass mold and carbonized at 1000 °C, in O_2_‐saturated electrolyte; D) multi‐chronoamperometry (mCA) graph of tubular ChiSuc as a gas diffusion electrode (GDE) with O_2_ and N_2_ gassing. The ChiSuc precursor was frozen in the designed brass mold (Figure S5C, Supporting Information) and carbonized at 1000 °C. All experiments were conducted in 50 mm Na_2_SO_4_ as electrolyte set to pH 3.

**Figure** **7**A–C represent the linear sweep and cyclic voltammograms (LSV & CV) for ChiSuc_Br_1000 as an electrode in an oxygen‐saturated solution. The monolithic ChiSuc_Br_1000 was directly used as an electrode after inserting a titanium wire as a current collector into the carbon structure (Figure S5B, Supporting Information). Based on Figure 7A, the onset potential for oxygen reduction reactions (ORRs) is around 0.58 V having its peak at 0.15 V. The decline in recorded current after 0.15 V can be attributed to the lack of oxygen as the primary reagent since the solution was oxygen‐saturated before LSV with no continuous oxygen supply during the measurement. The onset potential for the second peak is around −0.6 V, corresponding to a hydrogen evolution reaction (HER) occurring at higher cathodic potentials,^[^ ^50^ ^]^ which indicates the high over‐potential for the undesired HER on ChiSuc_Br_1000. Increasing the potential sweep rate from 1 mV s^−1^ to 50 mV s^−1^ boosts the recorded current density, e.g, from 3.5 mA cm^−2^ to 10.5 mA cm^−2^ at −1.3 V. Also, the peak observed for ORRs at 1 mV s^−1^ disappeared, being dominated by the non‐Faradaic, or capacitive, current resulting from the electrical double layer (EDL) displacement at higher potential sweep rates.^[^ ^51^ ^]^ EDL forms on the electrode‐electrolyte interface, representing the electrode's available surface area. Such high capacitive current in comparison to Faradaic one relates to the high specific surface area (629 m^2^ g^−1^) of the electrode and its microporous characteristics (67% of V_total_). Similarly, the CV graphs at two sweep rates (Figure 7C) have a typical rectangular shape, especially between −0.3 and 1 V, being characteristic for an excellent reversible capacitive current.^[^ ^25^, ^52^ ^]^ These results demonstrate the high potential of ChiSuc_Br_1000 for serving as supercapacitors, especially when one takes into account the low salt concentration (50 mm) and different salt's type (Na_2_SO_4_) considered in this work.^[^ ^53^ ^]^ A slight increase in recorded current density at around 0.6 V can be seen, corresponding to ORRs, that is in agreement with the LSV graph (Figure 7A). Such low Faradaic current in comparison to capacitive one, especially at higher sweep rates, implies that the oxygen availability is the limiting factor for Faradaic ORRs since ChiSuc_Br_1000 represents a high specific surface area for reactions.

### 2.4 Monolithic Porous Carbons as Tubular Gas Diffusion Electrodes

To show the versatility of the synthesis method in tailoring geometry and to overcome oxygen solubility for ORRs, a brass mold was explicitly designed (Figure S5C, Supporting Information) and used for fabricating tubular freestanding carbons. Again, ChiSuc was selected as the precursor, frozen in a tubular mold, and carbonized at 1000 °C like ChiSuc_Br_1000, being named as ChiSuc_GDE. Figure S5D and E, Supporting Information shows the tubular ChiSuc composite after lyophilization and the tubular carbon after sintering, respectively.

**Figure 8:**
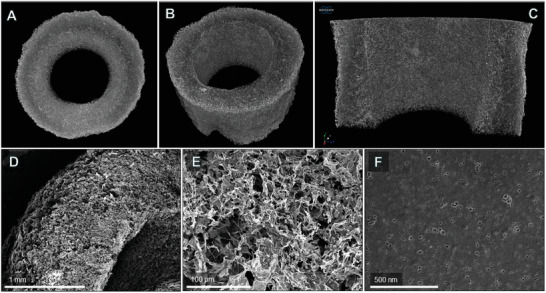
Micro‐CT 3D imaging (A‐C) and FESEM analysis (D‐F) of tubular freestanding carbons made of ChiSuc recipe, frozen in the designed brass mold (Figure S5C, Supporting Information) and carbonized at 1000 °C.

Micro‐CT analysis of ChiSuc_GDE (**Figure** **8**A–C) visualizes the tubular shape of the final product, with a uniform channel along the carbon length and a wall thickness of 1.2 ± 0.1 mm. Unlike ChiSuc_Br_1000 (Figure 6A), no radial freezing can be observed for ChiSuc_GDE (Figure 8D), representing a dense 3D macroporous network with no visible trend. This dense pore network implies that the ChiSuc precursor was frozen faster in the designed tubular mold (Figure S5C, Supporting Information) than the conventional one (Figure S5A, Supporting Information). The faster freezing rate comes from simultaneous cooling occurring from both sides (mold's shell and rod), which led to smaller ice‐templated pores (Figure 8E). Thus, the densest 3D pore network with the smallest ice‐templated macropores has been formed during the synthesis of ChiSuc_GDE. Silica‐templated mesopores formed during the etching‐out step on the macropores’ walls can be clearly seen in Figure 8F.

According to Table S4, Supporting Information, the BET surface area, micropore volume, and total pore volume for ChiSuc_GDE are 470 m^2^ g^−1^, 0.13 cm^3^ g^−1^, and 0.47 cm^3^ g^−1^, respectively. Unlike ChiSuc_Br_1000, the tubular carbon contains less BET surface area and micropores but a higher total pore volume. The higher total pore volume is because of a higher mesopore content, showing a fourfold higher peak height at 11.7 ± 0.9 nm than ChiSuc_Br_1000 (Figure S6, Supporting Information). This peak height is an indicator for silica removal efficiency during the etching‐out process, which strongly depends on the NaOH solution ingress into the 3D macroporous network of the final carbon as discussed earlier. Both the tubular shape and the smaller wall thickness of ChiSuc_GDE (≈one‐third of ChiSuc_Br_1000) promote NaOH solution ingress and silica removal during the etching‐out step. Conversely, the micropore volume is smaller for ChiSuc_GDE despite having smaller ice‐templated pores. This could be attributed to the restricted accessibility of the formed micropores during the N_2_‐analysis because of the isolated pore network. Therefore, ChiSuc_GDE is less microporous (28% of V_total_) than ChiSuc_Br_1000.

The tubular carbon was directly used as a GDE by inserting a spiral silver‐coated wire as the current collector and housing for gas inlet (Figure S5F, Supporting Information), representing an all‐carbon tubular GDE with neither adding any polymeric binder nor requiring further assembly steps like pressing on a rigid basis. Figure 7D represents the polarization curve for ChiSuc_GDE under N_2_ and O_2_ gassing and for ChiSuc_Br_1000 under O_2_ bubbling through a multi‐Chronoamperometry (mCA) experiment, where the average of Faradaic currents are plotted against the applied cathodic potentials. Accordingly, the onset potentials under O_2_ and N_2_ gassing are 0.58 V and −0.6 V for ORRs and HER, respectively; confirming the LSV and CV data achieved by ChiSuc_Br_1000. Moreover, much higher current densities were recorded for ChiSuc_GDE in comparison to ChiSuc_Br_1000 with solution bubbling, while the latter has a higher BET surface area and catalytic sites (Table S4, Supporting Information). This admits that the O_2_ solubility limits the electrochemical reactions on the electrode's surface despite having a high specific surface area and points out the crucial role of GDEs in providing efficient gas supply at catalytic sites. Moreover, a plateau is observed at −0.2 V for ChiSuc_Br_1000, showing the limited current density while solution bubbling. Conversely, the current density constantly increases for ChiSuc_GDE under oxygen gassing by increasing the applied potential, showing no limitation in the tested potential range. It is worth mentioning that the electrode potentials on the ChiSuc_GDE under O_2_ gassing are much lower than the applied potentials because of the significant ohmic drop (I * R) at high current densities. Based on Figure S7, Supporting Information, the corrected cathodic potential for an applied potential of −0.82 V with a recorded current density of 12.5 mA cm^−2^ is −0.15 V. This implies that even higher current densities can be achieved by further increasing the potential without any concern for HER.

## 3 Conclusion

In summary, we have introduced a novel approach with one‐step carbonization and activation for the production of N‐doped monolithic carbons through ice‐ and silica‐templating and using chitosan and sucrose as precursors. Notably, the effect of sonication on ice‐templating and silica‐templating was investigated, showing that sonicated samples lead to a lamellar and open ice‐templated macroporous structure. The 3D macroporous network plays an important role in both the meso‐ and microporosity achieved for the final carbon. Besides, all the carbons (sintered at 750 °C) possessed an N‐content varying from 2.47 ± 0.86 wt% to 3.43 ± 0.19 wt%, which were mainly in quaternary (≈64% area) and in pyridinic (≈36% area) forms. The SMX adsorption experiments illustrated the high adsorption capacity (up to 107 ± 2 mg g^−1^) of the sonicated samples considering their BET surface area of approximately 700 m^2^ g^−1^. However, a higher rate constant (pseudo‐first‐order) of 0.04 h^−1^ was achieved for [ChiSuc]_son._ than [Chi]_son._Suc (0.023 h^−1^) because of its larger ice‐templated macropore structure (Figure 3). In comparison, commercial GAC particles with a BET surface area of 1355 m^2^ g^−1^ resulted in an adsorption capacity of 135 mg g^−1^ and a rate constant of 0.016 h^−1^ under similar experimental conditions. A change of mold materials from PP to brass altered the freezing direction from longitudinal to radial, being favorable for solution ingress toward the inner parts of the monolith. The micro‐ and mesoporosity of the final products were tunable from a micropore‐dominated carbon (ChiSuc_Br_1000) to mesopore‐dominated ones (ChiSuc & ChiSuc_GDE) with the same precursors and silica content depending on the mold's material, type, and sintering temperature. Moreover, we demonstrated the functionality of as‐synthesized carbons for electrochemical purposes. The LSV results revealed the activity of carbons for ORRs while showing a sluggish behavior for the HER. Thus, the catalyst can exhibit up to 1 V kinetic overpotential without any hydrogen evolution interference . Also, CV data showed a rectangular shape with high current densities, implying their outstanding potential as supercapacitors . The flexibility of the synthetic method was shown by fabricating a tubular monolithic carbon, which was used as a GDE for ORRs. The mCA data indicated much higher Faradaic current densities because of the efficient supply of O_2_ at the electrode's surface by the gas channel. The introduced approach in producing N‐containing monolithic carbons opens up new possibilities of developing freestanding, binder‐free carbons, which can, particularly, be exploited in sustainable and hybrid processes for clean water supply.

## 4 Experimental Section

### Materials

High molecular weight (HMW) chitosan, sucrose (≥99%), acetic acid (≥99%), sodium sulfate (≥99%), sulfuric acid (99.5–99.7%), and conductive glue (Leit C) were purchased from Sigma‐Aldrich. Sulfamethoxazole (≥98%) and 40% colloidal dispersion of silicon dioxide (SiO_2_) in water with a density of 1.3 g mL^−1^ and a particle size of 20 nm were bought from Alfa Aesar. Sodium hydroxide (≥98 %) and charcoal granular activated carbon (GAC, about 2.5 mm, extra pure) were supplied by Carl Roth and Merck KGaA, respectively.

### Preparation of Precursors

First, a 3% chitosan stock solution was prepared by dissolving HMW chitosan (6 g) in deionized (DI) water (200 mL) with the help of acetic acid (2 mL). Second, 10 g of chitosan stock solution was weighed and poured into a 25 mL glass bottle. Third, DI water (10 mL) containing dissolved sucrose (0.3 g) was added to the bottle and mixed vigorously by hand. Afterward, the glass bottle was placed in an autoclave for 45 min at 120 °C, including 15 min reaction and 15 min for each of heating‐up and cooling‐down steps. Once the precursor cooled down to room temperature, colloidal silica (0.3g) was added; thus, the mass ratio of chitosan:sucrose:silica was 1:1:1. This precursor is named ChiSuc in which “Chi” stands for chitosan and “Suc” for sucrose.

To decrease the precursor's viscosity and better disperse the silica content added, the ChiSuc precursor was sonicated for 30 min in an ice‐bath after adding silica. This precursor is named [ChiSuc]_son._

An increase of free amino groups released from chitosan was aimed to accelerate chitosan and sucrose's reaction rate during autoclaving. Thus, another chitosan stock solution (3 wt%) was prepared in the same way but adding more acetic acid (8 mL). The prepared chitosan stock solution was also sonicated for 120 min in an ice‐bath. Both sonication and acetic acid content increase the degree of deacetylation in chitosan,^[^ ^35^ ^]^ and hence, the number of free amino groups available for the Maillard reaction.^[^ ^37^, ^38^ ^]^ The rest of the preparation was like above. This precursor is named [Chi]_son._Suc.

### Fabrication of Freestanding Carbons

All three precursors (ChiSuc, [ChiSuc]_son._, and [Chi]_son._Suc) were poured into polypropylene (PP) molds (ID = 10 mm), unless otherwise mentioned, and placed vertically on a freezing plate, which was constantly in contact with liquid nitrogen. The freezing plate was cooled down in liquid nitrogen 5 min prior to the freezing step. The frozen precursors were lyophilized for 24 h at −34 °C and 0.25 mbar, making ChiSuc, [ChiSuc]_son._, and [Chi]_son._Suc composites.Figure S2, Supporting Information represents a photograph of a ChiSuc composite frozen in PP molds after lyophilization. The composites were then carbonized at 750 °C, unless otherwise mentioned, under argon (Ar) atmosphere for 180 min. After carbonization, the initially added silica was etched‐out overnight using 3m NaOH at 80 C. The etched‐out carbons were washed several times with DI water until the washing solution's pH became neutral and then dried in a vacuum oven overnight.

### Characterization Methods

Tubular geometry, 3D macroporous network (ice‐templated pores), and mesopores (silica‐templated pores) were visualized through micro‐computed tomography (Micro‐CT, Bruker SkyScan 1272), scanning electron microscopy (SEM, Hitachi TM3000), and field emission scanning electron microscopy (FESEM, Hitachi S4800), respectively. Mesoporosity and microporosity were evaluated using N_2_‐physisorption analysis at 77 K by ASAP2020, Micrometrics. All the samples were degassed at 250 °C for 12 h before measurement. The specific surface area and pore size distribution (PSD) were calculated based on the Brunauer–Emmett–Teller (BET) method and the density functional theory (DFT), respectively, as recommended for micro‐ and mesoporous carbons.^[^ ^39^ ^]^ X‐ray photoelectron spectroscopy (XPS) was performed by an Ultra AxisTM spectrometer, Kratos Analytical, to evaluate the surface chemistry of the as‐synthesized carbons. Thermogravimetric analysis (TGA STA6000, Perkin Elmer) was employed to monitor both the carbonization of precursors at similar conditions (under an inert atmosphere (N_2_ flow) and a heating rate of 5 °C min^−1^) and the efficacy of the etching‐out process in removing silica particles by oxidizing the carbon (under O_2_ flow and a heating rate of 20 °C min^−1^). All the characterization methods were performed in duplicate, with the average value presented.

### Adsorption Experiments

Adsorption experiments were conducted at room temperature in 125 mL brownish glass bottles to avoid any possible photo‐degradation . 60 mL DI water containing 25 ± 1 mg L^−1^ SMX was poured into the bottles, and the adsorption started after adding a piece of as‐synthesized carbons or commercial GAC particles with the average mass of 10 ±1 mg. The concentration of SMX was measured through UV spectroscopy (Genesys 10S UV–Vis) at λ = 265 nm. The pH of the solution was not adjusted or controlled. All the adsorption experiments were performed in triplicates, with the average value reported. Error bars indicate the standard deviation.

### Electrochemical Experiments

Linear sweep voltammetry (LSV), cyclic voltammetry (CV), and multi‐chronoamperometry (mCA) were performed in an H‐cell with a three‐electrode configuration, using an Autolab potentiostat PGSTAT302N. A titanium mesh coated with platinum and a mercury sulfate 0.5M H_2_SO_4_ were used as a counter and a reference electrode, respectively. Cylindrical and tubular carbons based on the ChiSuc recipe and frozen in brass‐made molds were directly used as the working electrode without any further preparation steps like adding binder and immobilizing on a current collector. A titanium wire covered with Leit‐C as conductive glue was inserted inside the carbon monolith for electrical connection (Figure S5B, Supporting Information). 50 mm Na_2_SO_4_ in DI water, set to pH 3 with the help of H_2_SO_4_, served as both catholyte and anolyte being separated by a cation exchange membrane.

## Conflict of Interest

The authors declare no conflict of interest.

## Supporting information

Supporting Information

Supplemental Movie 1

## Data Availability

Research data are not shared.
