# Supplementary material for: Freestanding Nitrogen‐Doped Carbons with Hierarchical Porosity for Environmental Applications: A Green Templating Route with Bio‐Based Precursors
Source: Glob Chall. 2021 Aug 19;5(11):2100062. doi: 10.1002/gch2.202100062 (PMC8562066; doi:10.1002/gch2.202100062)
Supplement: Supplementary file 1 — Supporting Information [file GCH2-5-2100062-s001.pdf]

© 2021 Wiley-VCH GmbH

Global Challenges

Supporting Information

for *Global Challenges,* DOI: 10.1002/gch2.202100062

Freestanding Nitrogen-Doped Carbons with Hierarchical Porosity for Environmental Applications: A Green Templating Route with Bio-Based Precursors

*Mojtaba Mohseni, Nikolai Utsch, Christian Marcks, Kristof Demeestere, Gijs Du Laing, Süleyman Yüce, Robert G. Keller, and Matthias Wessling**

Freestanding Nitrogen-Doped Carbons with Hierarchical Porosity for Environmental Applications: A Green Templating Route with Bio-Based Precursors

Mojtaba Mohseni [a,b], Nikolai Utsch [a], Christian Marcks [a], Kristof Demeestere [b], Gijs Du Laing [b], Süleyman Yüce [a], Robert G. Keller [a], Matthias Wessling [a, c,*]

[a] RWTH Aachen University, Aachener Verfahrenstechnik – Chemical Process Engineering, Forckenbeckstr. 51, 52074 Aachen, Germany

[b] Ghent University, Department of Green Chemistry and Technology, Coupure Links 653, 9000 Ghent, Belgium

[c] DWI - Leibniz Institute for Interactive Materials, Forckenbeckstr. 50, 52074 Aachen, Germany

* Corresponding author: Prof.Dr-Ing Matthias Wessling

Email Address: manuscripts.cvt@avt.rwth-aachen.de (Matthias Wessling)

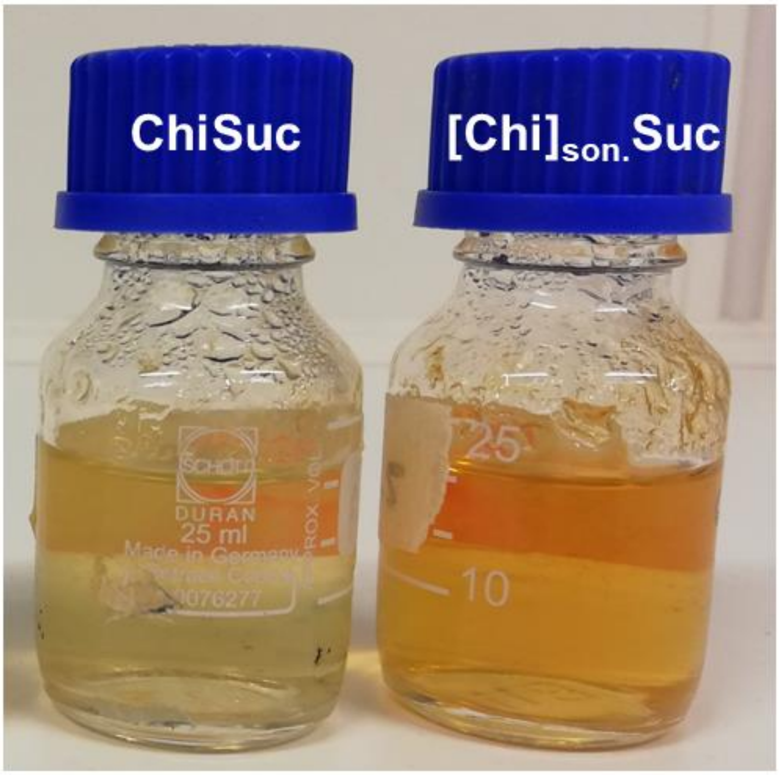

Figure S1: a photograph of ChiSuc and $[Chi]_{son.}$Suc precursors after autoclaving.

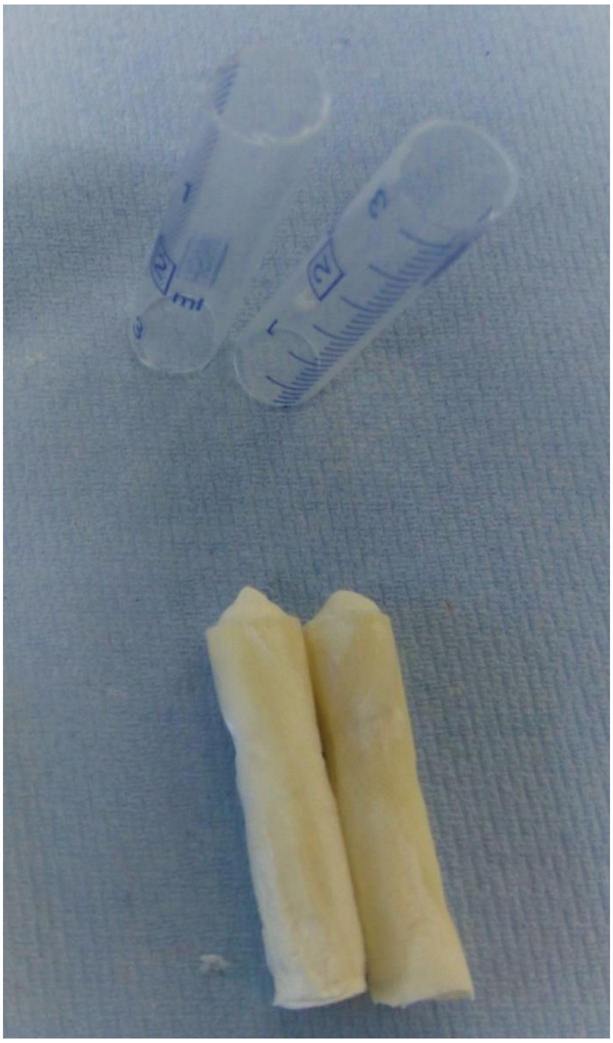

Figure S2: a photograph of monolithic ChiSuc composites frozen in plastic molds after lyophilization.

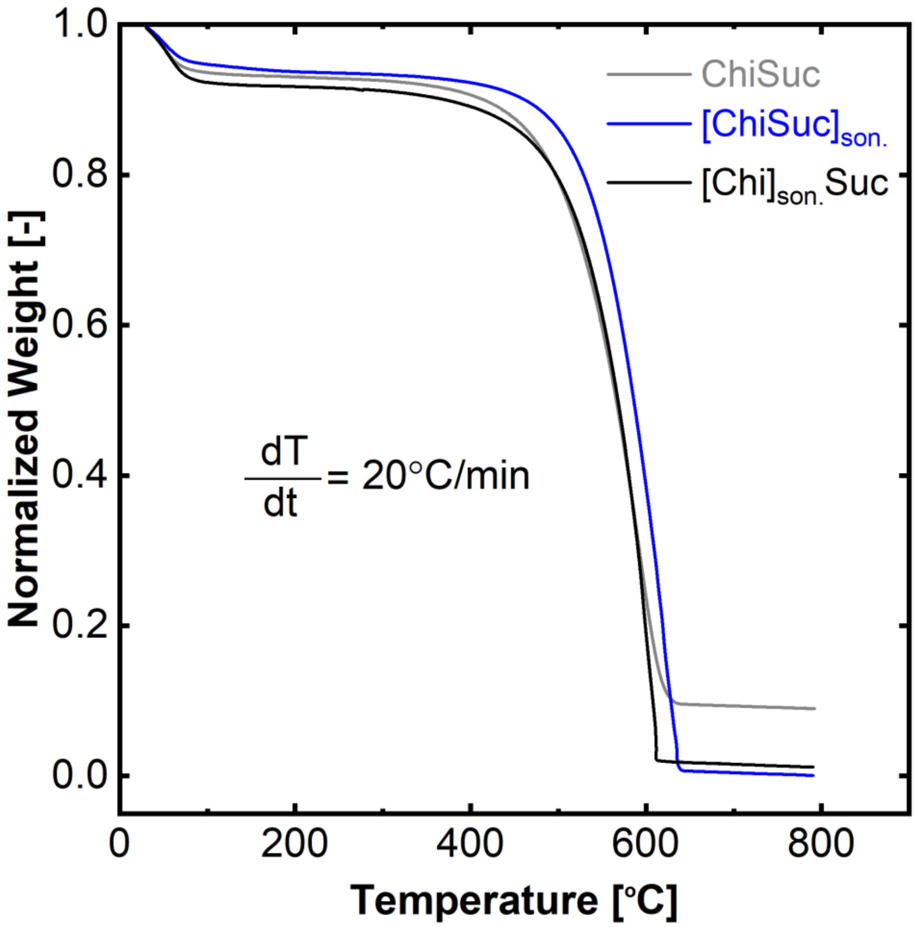

Figure S3: thermogravimetric analysis (TGA) of ChiSuc, $[ChiSuc]_{son.}$, and $[Chi]_{son.}Suc$ monolithic carbons performed under O2 gas from 30 °C to 850 °C (20 °C/min).

Table S1: raw data of cumulative pore volume (cm3/g), achieved based on the DFT model for different fabricated carbons.

Pore Width (nm)	Chisu_1st measur.	Chisu_2nd measur.	Schisu_1st measur.	Schisu_2nd measur.	Chisu-S_1st measur.	Chisu-S_2nd measur.
0.393	0.02529	0.02782	0.02768	0.0261	0.02518	0.02322
0.429	0.0474	0.05147	0.05253	0.04951	0.04784	0.04465
0.465	0.06553	0.07012	0.07375	0.06947	0.06724	0.06366
0.5	0.07915	0.0835	0.09072	0.08542	0.08282	0.07969
0.536	0.08815	0.09202	0.10314	0.09709	0.09427	0.09213
0.59	0.09308	0.09684	0.11142	0.10494	0.10192	0.10058
0.643	0.09479	0.09935	0.11605	0.10962	0.10618	0.10426
0.679	0.09488	0.10297	0.11837	0.11283	0.10835	0.10478
0.733	0.09488	0.11149	0.12038	0.11701	0.11042	0.10478
0.804	0.09488	0.12632	0.12405	0.12431	0.11433	0.10478
0.858	0.09671	0.13681	0.12989	0.13385	0.12014	0.10816
0.929	0.10235	0.13681	0.13709	0.14257	0.12644	0.11605
1.001	0.11161	0.13681	0.1443	0.14802	0.13195	0.12524
1.09	0.12231	0.13681	0.15125	0.15146	0.13748	0.13424
1.179	0.13175	0.13681	0.15839	0.15603	0.14452	0.14491
1.269	0.13839	0.13706	0.16542	0.16243	0.15263	0.155
1.358	0.14227	0.14072	0.17136	0.16895	0.15989	0.16198

1.483	0.14447	0.14532	0.17525	0.17347	0.16451	0.16559
1.591	0.14562	0.14853	0.17664	0.17493	0.16585	0.16559
1.716	0.14603	0.15004	0.17664	0.17493	0.16585	0.16559
1.859	0.14603	0.15014	0.17664	0.17493	0.16585	0.16559
2.002	0.14603	0.15014	0.17664	0.17493	0.16585	0.16559
2.162	0.14606	0.15014	0.17664	0.17493	0.16585	0.16559
2.341	0.14712	0.1514	0.17664	0.17493	0.16585	0.16559
2.52	0.14966	0.15437	0.17818	0.17552	0.1667	0.16559
2.734	0.15327	0.15835	0.18266	0.1792	0.17087	0.1702
2.949	0.15697	0.1619	0.18872	0.18458	0.17691	0.17597
3.181	0.1604	0.16532	0.19472	0.18985	0.18286	0.18136
3.431	0.16367	0.16883	0.19992	0.19421	0.18785	0.18619
3.699	0.16658	0.17161	0.20427	0.19771	0.19188	0.18978
4.003	0.16956	0.17465	0.20892	0.20159	0.19633	0.19423
4.325	0.17315	0.1782	0.21501	0.20687	0.20229	0.20006
4.664	0.17741	0.18251	0.22274	0.21381	0.21004	0.20746
5.04	0.18213	0.1872	0.23179	0.22208	0.21915	0.21616
5.433	0.18716	0.19223	0.24196	0.23147	0.22952	0.22618
5.88	0.19257	0.19762	0.25362	0.24238	0.24147	0.23767
6.344	0.19857	0.20369	0.26791	0.25612	0.25624	0.25179
6.845	0.20544	0.21069	0.28534	0.27333	0.27448	0.26986
7.399	0.21227	0.21746	0.30433	0.29192	0.29388	0.28865
7.988	0.2203	0.22551	0.32939	0.31621	0.31887	0.31223
8.632	0.23237	0.23789	0.3706	0.35678	0.35967	0.35133
9.311	0.24804	0.25374	0.42975	0.41521	0.41682	0.40585
10.061	0.26515	0.27087	0.50647	0.49156	0.48748	0.47105
10.866	0.28783	0.29282	0.60838	0.5956	0.58798	0.56623
11.723	0.32548	0.33126	0.70799	0.69579	0.71144	0.686
12.653	0.36647	0.37145	0.79393	0.78462	0.81971	0.7951
13.671	0.4026	0.40769	0.85322	0.84759	0.87596	0.85777
14.761	0.43024	0.43746	0.87195	0.86407	0.89997	0.88848
15.941	0.44471	0.45281	0.87563	0.86615	0.91043	0.90231
17.21	0.45096	0.45878	0.8803	0.87043	0.91756	0.91115
18.586	0.45546	0.46301	0.88359	0.87328	0.92163	0.91537
20.069	0.45984	0.46753	0.88458	0.87395	0.9238	0.91823
21.66	0.46329	0.47118	0.88514	0.87425	0.92559	0.92049
23.393	0.46556	0.47366	0.88559	0.87448	0.92686	0.92193
25.252	0.46732	0.47558	0.88589	0.87459	0.92766	0.92275
27.271	0.46876	0.47717	0.8861	0.87462	0.92828	0.92337
29.451	0.46964	0.47813	0.88624	0.87462	0.92868	0.92379
31.792	0.47029	0.47886	0.88634	0.87462	0.92904	0.92417
34.33	0.47092	0.47957	0.88646	0.87462	0.92948	0.92467
37.064	0.47138	0.48009	0.8866	0.87465	0.92982	0.9251
40.031	0.47173	0.48051	0.88682	0.87471	0.93011	0.92549
43.23	0.47196	0.48078	0.88702	0.8748	0.93037	0.92579
46.679	0.47198	0.48103	0.88723	0.87492	0.93067	0.92612
50.396	0.47198	0.48133	0.88755	0.87513	0.93113	0.9266
54.417	0.47198	0.48158	0.88787	0.87537	0.93166	0.9271
58.76	0.47198	0.48158	0.88812	0.87557	0.93218	0.92762
63.442	0.47198	0.48158	0.88859	0.87575	0.93263	0.92809
68.499	0.47198	0.48158	0.88891	0.87596	0.93315	0.92862
73.968	0.47198	0.48158	0.88912	0.87611	0.93351	0.92898
79.865	0.47198	0.48158	0.8893	0.87617	0.9338	0.92928
86.245	0.47198	0.48158	0.8895	0.87617	0.93412	0.92962
93.126	0.47198	0.48158	0.88965	0.87617	0.93427	0.92984
100.56	0.47198	0.48158	0.88972	0.87617	0.93427	0.92984
108.566	0.47198	0.48162	0.88972	0.87617	0.93427	0.92984
117.233	0.47198	0.48176	0.88972	0.87617	0.93427	0.92984
126.58	0.47198	0.48188	0.88972	0.87617	0.93427	0.92984

136.677	0.47199	0.482	0.88972	0.87617	0.93427	0.92984
147.596	0.47219	0.48216	0.88972	0.87617	0.93427	0.92984
159.355	0.47238	0.48231	0.88972	0.87617	0.93427	0.92984
172.079	0.47256	0.48245	0.88972	0.87617	0.93427	0.92984
185.804	0.47276	0.4826	0.88972	0.87617	0.93427	0.92984
200.619	0.47286	0.48268	0.88972	0.87617	0.93427	0.92984
216.632	0.47291	0.48272	0.88972	0.87617	0.93427	0.92984
233.913	0.47293	0.48274	0.88972	0.87617	0.93427	0.92984
252.57	0.473	0.48278	0.88972	0.87617	0.93427	0.92984

Table S2. peak assignments for C1(s), N1(s), and O1(s) of ChiSuc, $[ChiSuc]_{son.}$, and $[Chi]_{son.}Suc$ after sintering at 750 °C for three hours.

Peak	fraction of species [%]			Assignment
Binding energy [eV]	ChiSuc	$[ChiSuc]_{son.}$	$[Chi]_{son.}Suc$	
**C1**				
285 ± 0.0	83 ± 2.4	84.2 ± 1.7	84.5 ± 0.7	Sp^2 C-C or C-H [5-6]
286.4 ± 0.2	17 ± 2.4	15.8 ± 1.7	15.5 ± 0.7	C-O/ C-N [5-6]
**N1**				
398.7 ± 0.1	31.5 ± 1.8	37 ± 0.8	40.8 ± 3.4	Pyridinic[5, 7-8]
401.2 ± 0.1	68.5 ± 1.8	63 ± 0.8	59.2 ± 3.4	Quaternary[5, 7-8]
**O1**				
531.1 ± 0.3	18.5 ± 0.5	25.2 ± 1.1	14.6 ± 0.1	C=O[9]
532.6 ± 0.3	32 ± 0.1	41.4 ± 2.8	37 ± 1.7	C-O aliphatic[9]
533.9 ± 0.3	49.7 ± 0.4	33.5 ± 4	48.5 ± 1.9	C-O aromatic [9]

Table S3: kinetic constants of different carbons used for adsorption experiments with [SMX]0=25±1 mgL-1 based on the pseudo-first-order kinetic model: $Q_t = Q_e * (1 - e^{-k1*t})$.

Carbon	BET surface area [m^2/g]	V_{micro}/V_{total} [%]	Q_e [mg/g]	k1 [1/h]	R^2
ChiSuc	524 ± 12	31	53 ± 2	0.036 ± 0.009	0.97
$[ChiSuc]_{son.}$	684 ± 5	18	106 ± 2	0.040 ± 0.004	0.99
$[Chi]_{son.}Suc$	703 ± 6	20	107 ± 2	0.023 ± 0.003	0.99
Commercial GAC	1355*	60	135 ± 2	0.016 ± 0.001	0.99

* n=1

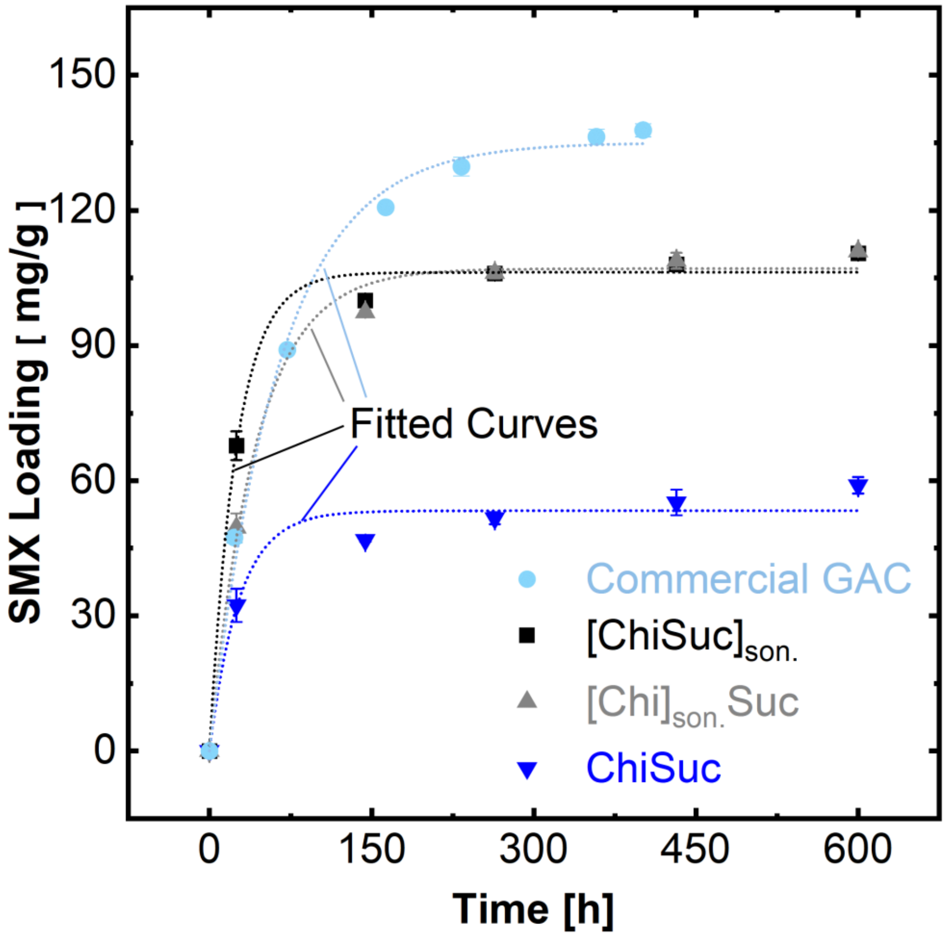

Figure S4: fitted curves to the experimental adsorption data for ChiSuc, [ChiSuc]son., [Chi]son.Suc, and commercial GAC based on the pseudo-first-order kinetic model with R^2 ≥0.97.

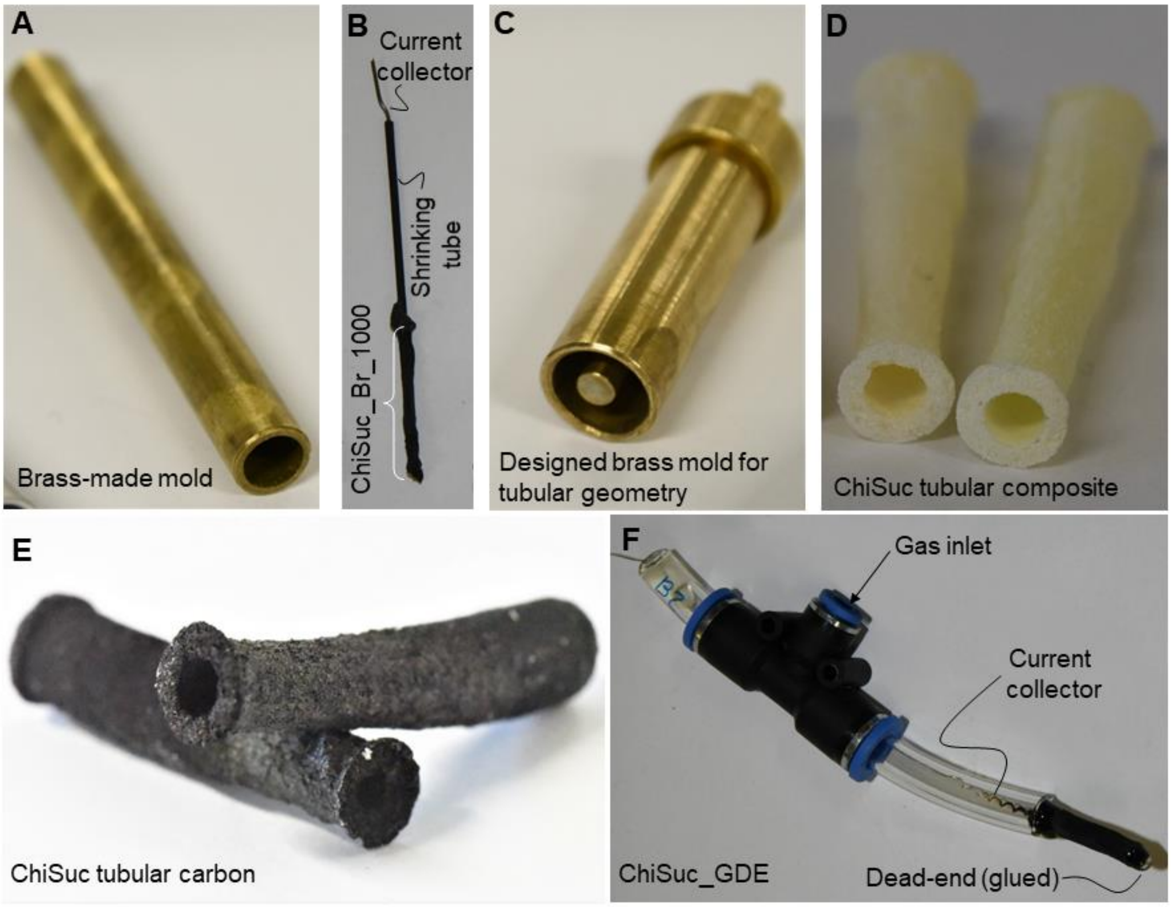

Figure S5: photographs of A) brass molds for cylindrical ChiSuc_Br_1000; B) freestanding ChiSuc_Br_1000 electrode prepared by inserting a titanium wire as a current collector; C) designed brass mold for tubular geometry; D) Tubular ChiSuc composites after lyophilization; E) carbonized ChiSuc with freestanding tubular geometry; F) tubular ChiSuc as a gas diffusion electrode (GDE) prepared by inserting a spiral silver-coated wire as a current collector and an IQS connector for gas supply.

Table S4: structural properties of ChiSuc recipe frozen in brass-made molds.

Carbon	S_{BET} (m^2/g)	V_{total} (cm^3/g)	V_{micro} (cm^3/g)
ChiSuc_Br_1000	629 ± 15	0.3 ± 0.01	0.20 ± 0.01
ChiSuc_GDE	470 ± 10	0.47 ± 0.01	0.13 ± 0.01

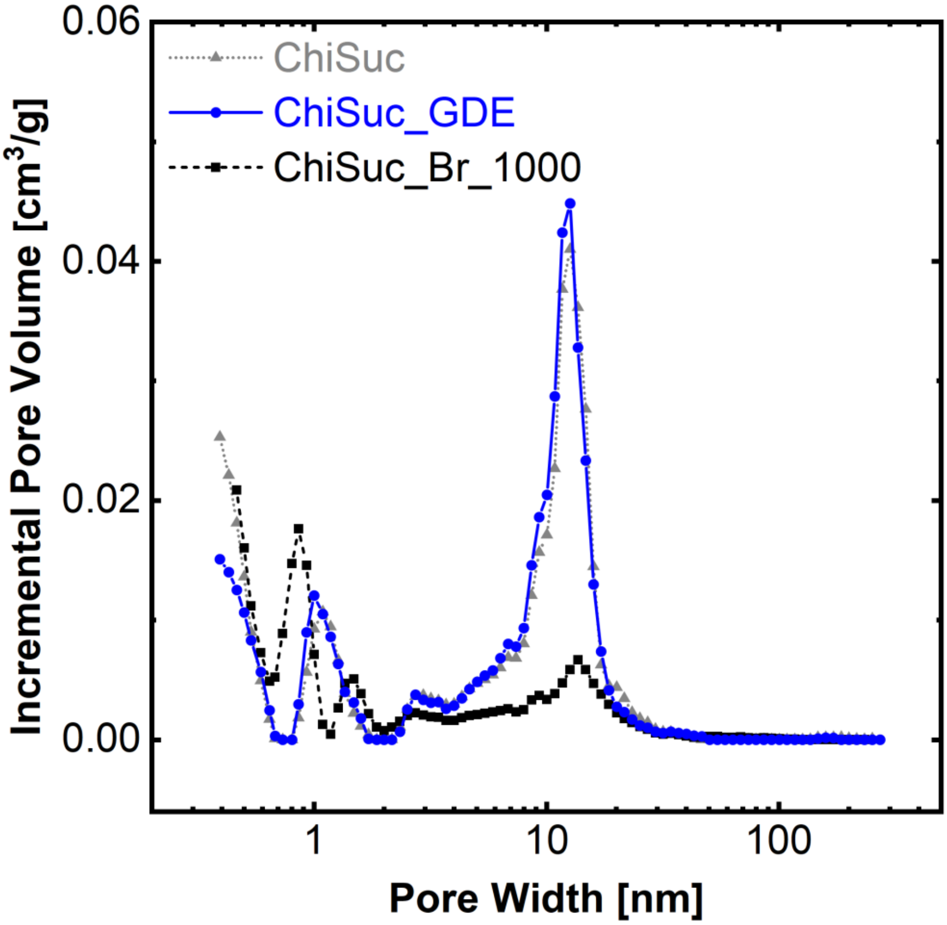

Figure S6: pore size distribution (PSD) based on DFT model for ChiSuc, ChiSuc_Br_1000, and ChiSuc_GDE

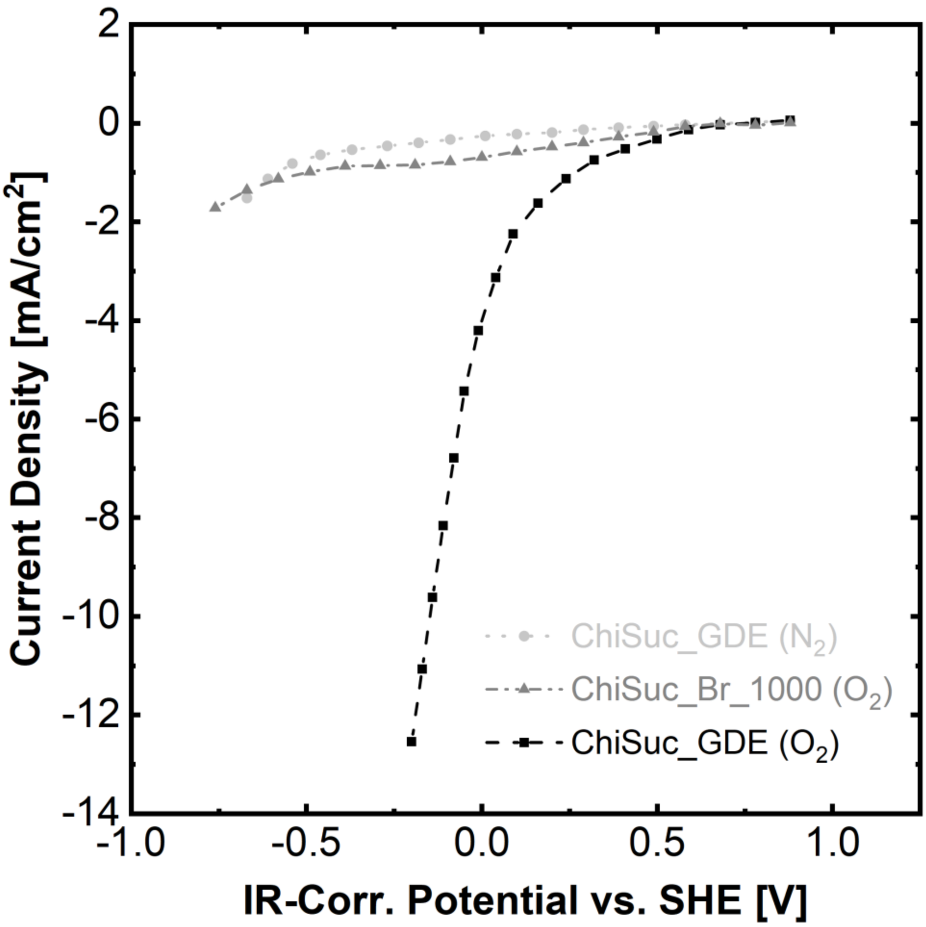

Figure S7: mCA analysis of ChiSuc_Br_1000 under O_2 bubbling and ChiSuc_GDE under O_2 and N_2 gassing with corrected potential according to the ohmic loss.

References

[1] Ellis, G. P. (1959). The maillard reaction. In *Advances in carbohydrate chemistry* (Vol. 14, pp. 63-134). Academic Press.

[2] Savitri, E., Juliastuti, S. R., Handaratri, A., & Roesyadi, A. (2014). Degradation of chitosan by sonication in very-low-concentration acetic acid. *Polymer Degradation and Stability*, *110*, 344-352.

[3] Nursten, H. E. (2005). *The Maillard reaction: chemistry, biochemistry, and implications*. Royal Society of Chemistry.

[4] Thommes, M., Kaneko, K., Neimark, A. V., Olivier, J. P., Rodriguez-Reinoso, F., Rouquerol, J., & Sing, K. S. (2015). Physisorption of gases, with special reference to the evaluation of surface area and pore size distribution (IUPAC Technical Report). *Pure and Applied Chemistry*, *87*(9-10), 1051-1069.

[5] Wohlgemuth, S. A., Vilela, F., Titirici, M. M., & Antonietti, M. (2012). A one-pot hydrothermal synthesis of tunable dual heteroatom-doped carbon microspheres. *Green chemistry*, *14*(3), 741-749.

[6] Choi, C. H., Park, S. H., & Woo, S. I. (2011). Heteroatom doped carbons prepared by the pyrolysis of bio-derived amino acids as highly active catalysts for oxygen electro-reduction reactions. *Green Chemistry*, *13*(2), 406-412.

[7] Stańczyk, K., Dziembaj, R., Piwowarska, Z., & Witkowski, S. (1995). Transformation of nitrogen structures in carbonization of model compounds determined by XPS. *Carbon*, *33*(10), 1383-1392.

[8] Khan, A., Goepel, M., Colmenares, J. C., & Gläser, R. (2020). Chitosan-Based N-Doped Carbon Materials for Electrocatalytic and Photocatalytic Applications. *ACS Sustainable Chemistry & Engineering*, *8*(12), 4708-4727.

[9] Smith, M., Scudiero, L., Espinal, J., McEwen, J. S., & Garcia-Perez, M. (2016). Improving the deconvolution and interpretation of XPS spectra from chars by ab initio calculations. *Carbon*, *110*, 155-171.

[10] Mohseni, M., Postacchini, P., Demeestere, K., Du Laing, G., Yüce, S., & Wessling, M. (2020). Freestanding PAC/CNT microtubes remove sulfamethoxazole from water through a temperature-assisted cyclic process. *Journal of Hazardous Materials*, *392*.

[11] Nielsen, L., Biggs, M. J., Skinner, W., & Bandosz, T. J. (2014). The effects of activated carbon surface features on the reactive adsorption of carbamazepine and sulfamethoxazole. *Carbon*, *80*, 419-432.

[12] Tonucci, M.C., Gurgel, L.V.A. and de Aquino, S.F., 2015. Activated carbons from agricultural byproducts (pine tree and coconut shell), coal, and carbon nanotubes as adsorbents for removal of sulfamethoxazole from spiked aqueous solutions: Kinetic and thermodynamic studies. *Industrial crops and products*, *74*, pp.111-121.
